# Brachial neuropathy 22 years after radiation therapy for fibrosarcoma: a case report

**DOI:** 10.4076/1757-1626-2-6838

**Published:** 2009-09-15

**Authors:** Sammy Al-Benna, Cornelius Schubert, Hans Ulrich Steinau, Lars Steinstraesser

**Affiliations:** Department of Plastic and Reconstructive Surgery, Soft Tissue Tumour Reference Centre, BG University Hospital Bergmannsheil, Ruhr University BochumBuerkle-de-la Camp Platz 1, 44789 BochumGermany

## Abstract

This case report presents a 56-year-old man with right upper limb weakness which arose 22 years after initial local radiation treatment for a grade III fibrosarcoma. Nerve conduction studies revealed impairment of all three major upper limb nerves compared with the left, with particular impairment of the median and ulnar nerves in the most fibrotic area that had been irradiated. In addition, the patient received multiple courses of chemotherapy. The occurrence of radiation-induced brachial plexopathy should be considered in patients presenting with limb pain or weakness even many years after radiation therapy.

## Introduction

1-3% of all adult soft tissue sarcomas are fibrosarcomas. The fact that soft tissue sarcomas constitute 1% of all adult malignancies underlines the rareness of fibrosarcomas. They most commonly occur in middle-aged and older adults with no sex-related differences. There are no specific predisposing factors, although some fibrosarcomas may develop after previous therapeutic irradiation. The tumours tend to be located in the deep soft tissues of extremities, trunk, head and neck and classically present as a well defined mass and cause local symptoms due to compression and invasion of local structures. The major prognostic factors include grade, tumour size and depth from the surface. 9-63% of all patients suffer from metastases which commonly occur in lungs and bones and rarely in lymph nodes. The overall 5-year-survival rate ranges between 39% and 54% and this emphasises the aggressiveness of this tumour.

## Case presentation

A 56-year-old retired Caucasian male presented in 2008 with weakness and discomfort which progressed over 3 months to involve his entire right forearm and hand. The symptoms were present at rest or with activity, and there were no exacerbating or releiving factors. He noted intermittent paresthesias of his entire hand with sensory loss in the lateral 3 and a half fingers of his right hand and slowly progressing arm weakness.

In 1985 he received neoadjuvant chemotherapy with Farmorubicin (total dose: 1200 mg) and Holoxan (total dose: 100 g) prior to surgical ablation of a G3 fibrosarcoma with clear margins in his right arm. In 1986 he underwent postoperative radiotherapy to a dose of 60 Gy in 30 fractions. In 1991, he developed a local recurrence and again received neoadjuvant chemotherapy with Farmorubicin (total dose: 1200 mg) and Holoxan (total dose: 100 g) followed by radical surgical ablation. This operation included partial resection of the biceps brachii, brachialis and triceps brachii. He was treated with further radiotherapy at single doses of 2 Gy up to a total dose of 20 Gy, followed by adjuvant chemotherapy with Farmorubicin (total dose: 300 mg) and Holoxan (total dose: 50 g). In 1997, he presented with a pathological fracture of his right humerus which was treated with an intramedullary nail.

Physical examination demonstrated slightly reduced radial and ulnar pulses compared to the left. Findings on a cranial nerve examination were normal with no Horner’s syndrome. Motor examination showed generalised M3 motor function of his right for earm and hand in contrast to normal M5 motor function of his contralateral upper limb. There was non-pitting oedema throughout his right forearm and dorsum of his hand. Biceps and triceps reflexes were reduced. Findings on a sensory examination demonstrated global superficial cutaneous S3 sensation in the forearm and hand with S2 senation in the lateral three and a half fingers of his hand and thenar eminence. Electrophysiologic studies were performed to evaluate the brachial plexus. Nerve conduction studies revealed reduced amplitude of median, ulnar and radial nerves compared with the left (Table 1). Needle electromyograhy (Table 2) showed large polyphasic motor unit potentials in the muscles innervated by the superior, middle and inferior trunks of the brachial plexus with no abnormal spontaneous activity or myokymic discharges. Magnetic resonance imaging (MRI) of the right arm showed fibrotic infiltrate and volume loss in the right arm and nodular ill-defined median, radial and ulnar nerves with no focal masses, consistent with postradiation changes. The clinical findings and supporting investigations indicated radiation-induced neuropathy involving the median, radial and ulnar nerves of the right upper limb. These injuries occurred within the field of prior radiation therapy. The patient was treated with compression therapy and physiotherapy. Surgical decompression was discussed, however, the patient elected to continue with conservative treatment only.

**Table 1.** Nerve conduction studies: summary of sensory nerves

Nerve	Site	Variable	Right	Left	Normal
Median	Digit 2	Amplitude (μV)	13.1	22.7	> 15.0
		Distal latency (ms)	3.4	2.8	< 3.6
Ulnar	Digit 5	Amplitude (μV)	14.3	19.2	> 15.0
		Distal latency (ms)	2.7	2.9	< 3.1
Radial	Anatomical snuffbox	Amplitude (μV)	20.9	NR	> 14.0
		Distal latency (ms)	4.1		< 2.7
Lateral antebrachial	Forearm	Amplitude (μV)	NR	9.3	
		Distal latency (ms)		2.1	
Medial antebrachial	Forearm	Amplitude (μV)	NR	NR	
		Distal latency (ms)		NR	

mV = millivolts, ms = milliseconds, m/s = metres per second, μV = microvolts, NR = no response.

**Table 2.** Electromyographic needle examination

Side	Muscle	Nerve	Root	INS	FIBS	PSW	FAS	AMP	DUR	CONFIGURATION		REC PAT	REC INT
R	1st Dor Int	Ulnar	C8-T1	Nml	1+	2+	0	Nml	Nml	Di/Tri	Phasic	Full	Normal
R	APB	Median	C8-T1	Nml	1+	2+	0	Nml	Nml	Di/Tri	Phasic	Full	Normal
R	Abd Dig Min	Ulnar	C8-T1	Nml	1+	2+	0	Nml	Nml	Di/Tri	Phasic	Discrete	Dec
R	Biceps	Musc	C5-6	Nml	0	1+	0	Nml	Nml	Di/Tri	Phasic	Full	Normal
R	Brachialis	Musc	C5-6	Nml	0	0	0	Nml	Nml	Di/Tri	Phasic	Full	Normal
R	Cerv para Low	Rami	C6-7	Nml	0	0	0	Nml	Nml	Di/Tri	Phasic	Full	Normal
R	Cerv para Up	Rami	C5-6	Nml	0	0	0	Nml	Nml	Di/Tri	Phasic	Full	Normal
R	Deltoid	Axilla	C5-6	Nml	0	0	0	Nml	Nml	Di/Tri	Phasic	Full	Normal
R	Ext Car Rad	Radial	C7-8	Nml	2+	2+	0	Nml	Nml	Di/Tri	Phasic	Dec	Normal
R	Ext Dig Com	Radial	C7-8	Nml	1+	2+	0	Nml	Nml	Di/Tri	Phasic	Dec	Normal
R	Ext Ind Pro	Radial	C7-8	Nml	1+	2+	0	Nml	Nml	Di/Tri	Phasic	Dec	Normal
R	Flex Car Rad	Median	C6-8	Nml	2+	3+	0	Nml	Nml	Di/Tri	Phasic	Discrete	Dec
R	Flex Dig Prof	Ulnar	C8-T1	Nml	1+	2+	0	Nml	Nml	Di/Tri	Phasic	Full	Nml
R	Flex Car Uln	Ulnar	C8-T1	Nml	1+	2+	0	Nml	Nml	Di/Tri	Phasic	Full	Nml
R	Infraspinatus	Supra	C5-6	Nml	0	0	0	Nml	Nml	Di/Tri	Phasic	Full	Normal
R	Lat triceps	Radial	C6-7-8	Nml	0	0	0	Nml	Nml	Di/Tri	Phasic	Full	Normal
R	LongHdTricep	Radial	C6-7-8	Nml	0	0	0	Nml	Nml	Di/Tri	Phasic	Full	Normal
R	PalmarisLong	Median	C8-T1	Nml	1+	1+	0	Nml	Nml	Di/Tri	Phasic	Full	Normal
R	Pronator teres	Median	C6-7	Nml	2+	2+	0	Nml	Nml	Di/Tri	Phasic	Discrete	Dec
R	Rhomboids	DorsS	C5	Nml	0	0	0	Nml	Nml	Di/Tri	Phasic	Full	Normal
R	Serrat Ant	LnTho	C4-6	Nml	0	0	0	Nml	Nml	Di/Tri	Phasic	Full	Normal
R	Supraspinatus	Supra	C5-6	Nml	0	0	0	Nml	Nml	Di/Tri	Phasic	Full	Normal
R	Trapezius	Spin	C3-4	Nml	0	0	0	Nml	Nml	Di/Tri	Phasic	Full	Normal

*Ins* = insertional activity, *Fibs* = fibrillation potentials, 0 = absent, 1+ minimal to 4+ maximal, *PSW* = positive sharp wave, *Fas* = fasciculation, *Amp* = amplitude, *Dur* = duration, *Rec Pat* = recruitment pattern, *Rec Int* = recruitment interval, *Nml* = normal, *Dec* = decreased, *1st Dor Int* = first dorsal interosseus, *APB* = abductor pollicis brevis, *Abd Dig Min* = abductor digiti minimi, *Cerv para* = cervical paraspinal, *Ext Car Rad* = extensor carpi radialis, *Ext Dig Com* = extensor digitorum communis, *Ext Ind Pro* = extensor indicis proprius, *Flex Car Rad* = flexor carpi radialis, *Flex Dig Prof* = flexor digitorum profundus, *Flex Car Uln* = flexor carpi ulnaris,* Lat triceps* = lateral head pf triceps, *LongHdTricep* = long head of triceps, *Serrat Ant* = serratus anterior.

## Discussion

Radiation therapy is a common adjunctive modality used in the treatment of soft tissue sarcomas after surgical ablation. Although radiotherapy has shown benefit in reducing sarcoma recurrence and improves survival, it has associated risks. Complications of radiation therapy may involve the skin, lymphatics, peripheral vasculature, and peripheral nervous system. The most common symptoms in the arm include skin changes, reduced mobility of the arm, or secondary lymphedema, and they usually occur after local high-dose radiation. A less common but potentially more serious complication is radiation-induced brachial neuropathy. The clinical manifestations include gradually progressive paresthesias and sensory loss, weakness and atrophy, and pain. Symptom onset ranges from 1 month to 18 years after radiation exposure [1]. The main theme of this case is the progressive late polyneuropathy after two courses of therapeutic radiotherapy. Hand function has progressively deteriorated. As the first course of radiotherapy was in 1986, 22 years ago, and the second course in 1991, 17 years ago, this case illustrates the severe and progressive late effects many years after radiotherapy. Multiple surgeries, the humeral fracture and three courses of chemotherapy may also have contributed to the neuropathy. The key evidence in favour of radiation-induced injury is the fibrosis located within the field of prior radiation therapy. During sarcoma surgeries, there was no evidence of infiltration of any major nerves and MRI of his right arm showed fibrotic infiltrate and volume loss in the right arm with nodular ill-defined median, radial and ulnar nerves with no focal masses, consistent with postradiation changes. Chemotherapy is known to increase the the risk of neuropathy. Olsen et al. observed that patients receiving additional chemotherapy have a higher incidence of neuropathy than patients receiving radiotherapy alone [2]. The development of fibrosis is a gradual process with a median interval between radiotherapy and occurrence of brachial neuropathy ranging between 1-4 years [3]. In most patients, electrophysiologic studies show abnormal motor and sensory nerve conduction and large motor unit potentials with reduced recruitment. Myokymic discharges are found in up to 63% of patients and are helpful in distinguishing radiation-induced neuropathies from neoplastic neuropathies [4]. Predisposing factors for the development of radiation-induced neuropathy include the total radiation dose and dose per fraction with a total tolerance dose of 60 Gy and with doses greater than 2 Gy per fraction [5]. Size and localisation of the irradiated area also play a crucial role. In this case the total dose given in two therapies was 80 Gy and thus exceeded the accepted tolerance dose of 60 Gy [5]. The dose per fraction was 2 Gy and within accepted tolerance limits [5]. Side effects of radiotherapy can be subdivided into early and late effects. Early effects occur two days after nerve irradiation and include bioelectrical alterations, enzyme changes, abnormal microtubule assembly and altered vascular permeability. The late effects occur between 1 year and decades post-irradiation and they can be split into two phases. The first phase includes changes in electrophysiology and histochemistry of neurones and glial cells and the second phase includes fibrosis of the tissue surrounding the nerves. Indirect ischaemic damage due to microvascular injury also harms neurones and glial cells. It is important though to stress that the major damage caused in neurones, DNA damage, takes place immediately at the instant of ionization. As the mitotic rate of these cells is very slow, the damage is normally not expressed until the cell tries to divide. Therefore, this damage is latent for periods ranging from days to decades. These changes lead to the the clinical manifestations that this patient suffered from, including gradually progressive paresthesias, hypoaesthesias and dysaesthesias, paresis and atrophy, hyporeflexia, pain and oedema. Adverse complications in bones include fracture, osteoradionecrosis with potential creation of sequestra. It is always important in individual patients to balance the aggressiveness of the radiotherapy with its potential and severe complications against the risk of potential recurrences. The occurrence of radiation-induced brachial plexopathy should be considered in patients presenting with upper extremity pain or weakness even many years after radiation therapy. The course of the radiation-induced neuropathy is one of steady progression or stabilisation in 90% of patients, although cases of improvement have been reported [7-9]. No treatment is available to reverse or improve the nerve injury, although surgical intervention, such as neurolysis or neurolysis with omental grafting, has been performed in some patients with variable improvement in symptoms [10,11]. Radiation-induced brachial plexopathy is an uncommon complication of radiation treatment of fibrosarcoma, which can occur years after the initial exposure. The risk of these complications is expected to decrease with the use of modern state-of-the-art radiation techniques. In patients with symptoms, including pain, sensory loss, or weakness, who have received prior radiation, injury to nerves should be considered.

This case report reminds us that *de novo* symptoms and signs of radiation-induced brachial plexopathy can arise even after twenty two years of satisfactory upper limb function. It is important for clinicians to inform patients that complications of radiation therapy, which may involve the skin, lymphatics, peripheral vasculature, and peripheral nervous system can arise very late. The most common complications in the first year post radiation therapy include skin changes, reduced mobility and secondary lymphedema but the onset of radiation-induced neuropathy can occur as a complication from early to very late time periods.

**Figure 1.**
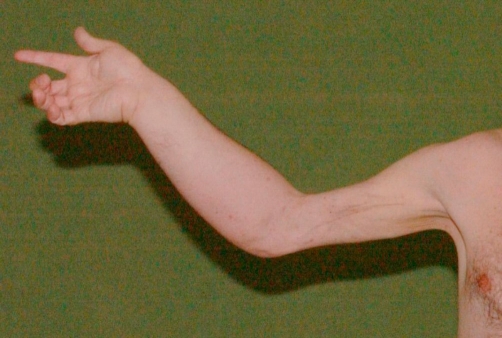
The clinical picture of patient’s right upper limb showing marked changes in appearance including fibrosis of the arm and oedema of the forearm 22 years after initial radiotherapy.

**Figure 2.**
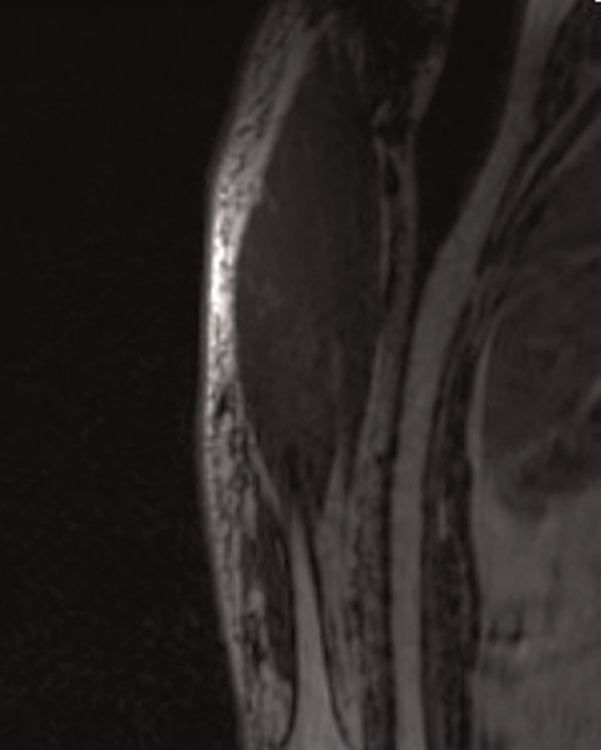
Magnetic resonance imaging demonstrating showed fibrotic infiltrate from radiotherapy.

**Figure 3.**
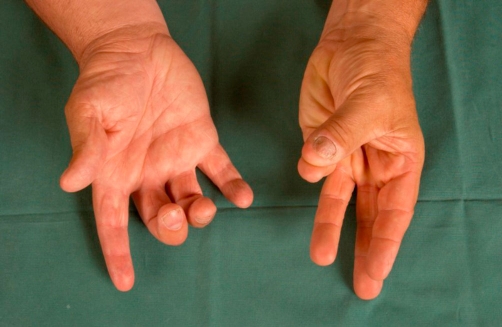
22 years after initial radiotherapy there is reduced function of the right hand and particularly of the right thumb which is unable to oppose against the little finger.
